# The adequacy of workplace accommodation and the incidence of permanent employment separations after a disabling work injury or illness

**DOI:** 10.5271/sjweh.4149

**Published:** 2024-04-01

**Authors:** Cameron A Mustard, Christa Orchard, Kathleen G Dobson, Nancy Carnide, Peter M Smith

**Affiliations:** 1 Institute for Work & Health, Toronto, Ontario, Canada.; 2 Dalla Lana School of Public Health, University of Toronto, Toronto, Ontario, Canada.

**Keywords:** cohort study, disability, work injury

## Abstract

**Objective:**

This study aimed to estimate the influence of the adequacy of employer accommodations of health impairments in predicting permanent separation from the employment relationship in a cohort of workers disabled by a work-related injury or illness.

**Methods:**

The study used data from a retrospective, observational cohort of 1793 Ontario workers who participated in an interviewer-administered survey 18 months following a disabling injury or illness. The relative risks (RR) of a permanent employment separation associated with inadequate employer accommodations were estimated using inverse probability of treatment weights to reduce confounding.

**Results:**

Over the 18-month follow-up, the incidence of permanent separation was 30.1/100, with 49.2% of separations related to health status. Approximately 51% of participants experiencing a separation were exposed to inadequate workplace accommodations, compared to 27% of participants in continuing employment. The propensity score adjusted RR of a health-related separation associated with inadequate accommodation was substantial [RR 2.72; 95% confidence interval (CI) 2.20–3.73], greater than the RR of separations not related to health (RR 1.68; 95% CI 1.38–2.21).

**Conclusion:**

Incidence of permanent separation in this cohort of Ontario labor force participants was approximately two times more frequent than would be expected. The adequacy of employer accommodation was a strong determinant of the risk of permanent separation. These findings emphasize the potential for strengthened workplace accommodation practices in this setting.

Employment separation, whether voluntary or involuntary, is common among working adults in the developed economies. In any given year, approximately 6% of Canadian workers aged 15–64 years age will voluntarily separate from an employment arrangement, and 4% will experience job loss or involuntary employment separation (1). The reasons for voluntary employment separation are varied and highly dependent on age. These include decisions to seek educational opportunities, family care responsibilities, dissatisfaction with current employment, retirement, and decisions to withdraw from employment due to illness or disability (involuntary employment separations may also arise from illness or disability). Among those aged 25–54 years, the incidence of leaving employment due to illness or disability in Canada is approximately 6 per 1000 working adults, representing approximately 20% of voluntary employment separations.

Empirical studies of health-related employment separation (HRES) are dominated by cohorts of workers with specific conditions, such as arthritis, mental health disorder or traumatic injury (2–6). However, observational population-based studies of HRES are less frequently reported. A UK study has estimated an incidence of HRES of 13% among approximately 10 000 male participants aged 24–70 years, based on a recall of lifetime work histories (7). Dominant conditions responsible for HRES in this study included musculoskeletal disorders (33%), mental health problems (25%) and injuries (12%). A more recent UK cohort study of 4900 older workers aged 50–64 years estimated an incidence of HRES of 5.8/100 workers over a 24-month follow-up period between 2014 and 2016 (8). In a Danish cohort of 5600 workers aged 18–67 years and employed in 20 industrial or 19 public sector workplaces in 2002, the incidence of employment separation at a 24-month follow-up survey was 17.3%, of which approximately one-quarter – 4% of the overall cohort – reported the separation was attributed to health conditions (9). A Dutch cohort of 9160 workers aged 45–64 years reported an incidence of employment separation of 18.8% over a seven-year follow-up period, of which approximately 20% were attributed to chronic health conditions (10). Finally, in an analysis pooling seven prospective cohort studies from Finland, France, the UK, and USA, 99 000 participants with a mean age at baseline of 48 years were followed for 7–18 years (11). Over 834 000 person years at risk, there were 50 000 work exits, of which 14% were health-related.

At the population level, a portion of HRES can be attributed to health impairments arising from occupational exposures. In addition to substantial regulatory attention in the developed economies to prevent work-related illness and injury, many jurisdictions place emphasis on the obligations of employers to provide employment security to workers experiencing a disabling work-related injury or illness. Two decades of empirical research has evaluated the effectiveness of workplace accommodations in maintaining employment of workers with health impairments or enabling return-to-work following a disabling work-related injury or illness (12–15). These accommodations may include changes to a work schedule, the modification of job tasks, changes to the pace or speed of work or changes to equipment and tools (16, 17). They will also typically involve return-to-work planning discussions involving both the worker and the employer and optimally include the designation of an employer representative to coordinate and supervise the return-to-work process (18). This evidence has supported the adoption of workplace policies and practices (19), the implementation of disability insurance benefit innovations to support workplace accommodation practices (20), and in some settings implementing regulatory requirements obligating employers to re-employ a disabled worker and to provide accommodations to enable return to work.

Considerable research in the area of disability management has focused on identifying employer practices that improve return-to-work outcomes among disabled workers (21). The perspective of this study is different; in this observational cohort study of workers disabled by a work-related injury or illness, we aim to understand the impact of employer accommodations of workers’ health impairments on the risk of permanent separation from the employment relationship. Specifically, we estimate the employment separation rate in a cohort of disabled workers, distinguishing between voluntary and involuntary separations and separations that are related to the workers’ health. Further, in this observational cohort of workers disabled by a work-related injury or illness, we use propensity score weighting to assess the influence of the adequacy of employer accommodations of health impairments in predicting permanent separation from the employment relationship. This method has the benefit of matching the distribution of measured confounding variables of the group of injured workers who had adequate accommodation for their injury/illness to the group of injured workers with inadequate accommodation.

## Methods

### Study design

The study design is a retrospective cohort study of workers disabled by a work-related injury or illness. The exposure of interest – the adequacy of employer accommodations – and the outcome of interest – the incidence of permanent employment separation – were obtained from interviewer-administered interviews approximately 18 months following the disabling work-related injury or illness.

### Setting

The setting of this study is Ontario, Canada. In Ontario, there are approximately 7 million labor force participants, with ~70% employed by organizations with a mandatory obligation to obtain worker disability insurance coverage for work-related injuries and illnesses from the Ontario Workplace Safety & Insurance Board (WSIB). The WSIB is a publicly administered, single-payer workers’ compensation insurance authority. For entitled workers who have experienced a work-related injury or illness that requires an absence from work, the WSIB administers benefits that cover the provision of wage replacement and medical care service benefits. In 2018, out of the ~200 000 compensation claims administered by the WSIB, ~33.5% resulted in one or more days absent from work.

### Participants

The Ontario Life After Work Injury Study (OLAWIS) consolidates information from two cohorts of workers in Ontario, Canada, who were disabled by a physical work injury or illness and received wage replacement benefits. Both cohorts were comprised of workers ≥18 years, employed by an insured employer, and who had experienced a physical work-related injury or occupational disease that resulted in a WSIB accepted lost-time compensation claim. To obtain sufficient representation of more serious and complex disability episodes, disabled workers with longer wage replacement durations of ≥3 months were oversampled. Participants with wage replacement benefit durations of one day to three months, representing 85% of all accepted claimants in this setting, comprised 41% of the combined sample.

The first OLAWIS study (OLAWIS1) had a pre-specified objective of recruiting 1200 participants. Participant recruitment occurred between June 2019 and March 2020, among workers disabled by a work-related injury or illness from January to October 2018. A sampling frame of 9745 eligible participants were selected by representatives of the WSIB, from which randomly sampled participants were contacted by WSIB representatives by telephone to obtain monthly quotas of claimants consenting to share their contact information. Of the 2816 claimants contacted, 1674 (59.4%) agreed to share their contact information. A survey services contractor completed interviews with 1132 claimants (40.1% of claimants contacted by the WSIB and 87.7% of claimants successfully contacted by the survey services contractor). Among participants, 358 (31.6%) were in the short-duration claim sample and 774 (68.3%) were in the long-duration claim sample. More details on the OLAWIS1 study cohort may be found elsewhere (22). The second study (OLAWIS2) had a pre-specified objective of recruiting 700 participants. Participant recruitment occurred over September to November 2021 among workers disabled by a work-related injury or illness in January or February 2020. Of the 7368 claimants in the sample frame for the OLAWIS2 cohort, a survey services contractor contacted 2309 claimants, of whom 121 (5%) were not eligible, 1488 (64%) declined the invitation to participate in an interview, and 700 (30%) accepted the invitation. Among OLAWIS2 participants, 395 (56.4%) and 305 (43.6%) were in the were in the short- and long-duration claim sample, respectively.

The present study included all cohort members of the OLAWIS1 (N=1132) and OLAWIS2 cohorts (N=700), totaling 1832 participants. The Health Sciences Research Ethics Board at the University of Toronto granted research approval (Protocols 37525 and 41560).

### Data sources

Information on permanent separation status, accommodations provided by the workplace and demographic and occupational characteristics were obtained from the interviewer-administered telephone questionnaire 18 months after the original injury or illness occurred. Additional information on the nature of injury was obtained from administrative records of work disability insurance benefits, linked to survey responses. Of OLAWIS1 and OLAWIS2 participants, 94% and 84%, respectively, consented to the linkage of administrative records to survey responses.

### Exposure measure: adequacy of accommodation

Approaches to defining the elements of effective workplace accommodation practices (17, 18, 23) have primarily described variation in accommodation practices within samples of employers. There have been limited application of these approaches in establishing benchmarks for minimally adequate policies and practices, and also in evaluating the relative effectiveness of specific accommodation practices in improving work disability outcomes (14, 24, 25).

We anchored the measure of accommodation adequacy to two aspects of the employer’s disability management practice, based on respondent self-report: (i) offer of modified work (participants were asked, “did the workplace offer modified or alternate duties to assist in returning to work?”), and (ii) return-to-work coordination (participants were asked, “did someone from your workplace help to coordinate the details of dates, activities and hours of your return to work?”). A total of 70.0% of participants reported an offer of modified work, and 60.1% reported return-to-work coordination.

For analyses, a binary variable was created to denote accommodation adequacy. Participants reporting a modified work offer and return-to-work coordination were classified as receiving adequate accommodation, while participants reporting a modified work offer and no coordination were defined as receiving inadequate accommodation. Additionally, participants reporting not receiving a modified work offer were stratified into two groups. On the assumption that recovery of function progresses quickly and that there are limited needs for accommodation in an important fraction of disabling work-related disorders, workers not offered modified work and who reported no substantial difficulties in return-to-work were defined as experiencing adequate accommodation. Workers who did not received an offer of modified work and who reported substantial difficulties in return-to-work were defined as experiencing inadequate accommodation. Two observations missing information necessary to classify accommodation status were excluded.

### Outcome measure: permanent separation from the at-injury employer

At the 18-month interview, claimants reported their current employment status: employed with the at-injury employer in the same job, employed with the at-injury employer in a different job, employed with a different employer than the at-injury employer, or not working. The last two categories were classified as permanent separations from the at-injury employer. Among those who separated from their at-injury employer, separations were classified as health-related versus not health related, and voluntary (the worker chose to separate from the employer) versus involuntary (ie, the employment contract ended, there was no work available, the employer dismissed or terminated the employee) based on information from question item response options classified at the time of interview (454 participants) and a subsequent classification of open-text responses (98 participants). Additionally, the review of open-text responses identified a misclassification of 78 participants who were not working at the time of interview but who had a continuing employment relationship with the at-injury employer (details available in the supplementary material, www.sjweh.fi/article/4149). A total of 39 permanent separations who could not be classified accurately to either the health-related or the voluntary/involuntary dimension and 32 participants who reported retiring in the 18-month period following the disabling injury/illness were excluded from the analysis of permanent separation and accommodation adequacy.

### Analysis

*Propensity weighting (inverse probability of treatment weighting).* In assessing permanent separation outcomes between workers who experienced adequate accommodation and those exposed to inadequate accommodation, confounding is likely if the distribution of baseline characteristics that affect the outcome differ between accommodation adequacy groups. In this context, it is likely that different socio-demographic and occupational characteristics have different propensity for receiving adequate accommodation and may also have differential likelihood of retaining permanent employment. Accordingly, we used inverse probability of treatment weighting (IPTW) using a propensity score to reduce the effects of potential confounding (15, 26). The IPTW create a pseudo population with a balanced distribution of measured covariates between the accommodation groups. Inverse probability of treatment weights each participant by the inverse of the probability of their accommodation status. Propensity score analyses have two underlying assumptions (27, 28). The first assumption is that the propensity score includes all potential confounding variables, while the second assumption is that each participant has a non-zero probability of receiving each level of the exposure.

The propensity score was derived using a logistic regression model, in which a binary variable denoting accommodation adequacy status was regressed on a series of independent variables. We included independent variables that were hypothesized or are known to be associated with accommodation adequacy and may be potential confounders of the relationship between accommodation adequacy and the likelihood of a permanent employment separation: worker sociodemographic characteristics (age, sex, highest level of education, immigration history, and urban/rural residence); at-injury workplace characteristics (union membership, firm size, economic sector, and permanent vs. temporary employment); the nature of the work-related injury and sample group membership (short and long duration). A multiple imputation procedure, based on 500 bootstrap samples and averaging across 20 imputation samples per bootstrap, was applied to observations missing information on the nature of injury. Inverse probability of treatment weights were then calculated using the propensity scores as the inverse of probability of adequate accommodation. Standard differences (SD) for each of the confounding covariates across those with and without adequate accommodation were examined before and after weighting, to ensure balance was achieved. The number exceeded a cutoff 0.1 SD were examined across imputations and bootstrapped samples before and after weighting.

Using the weighted sample, we estimated relative risks (RR) for permanent separation based on adequate accommodation exposure in the analytic sample of 1793 participants. We examined four exclusive permanent separation outcomes: (i) voluntary and not health related, (ii) voluntary and health-related, (iii) involuntary and not health related, (iv) involuntary and health related. RR for each of the four permanent separation outcomes relative to no permanent separation were calculated by dividing the estimated probability of each outcome among those without adequate accommodation by the estimated probability among those with adequate accommodation. We also calculated RR for two combined categories of permanent separation: (i) any health-related separation and (ii) any involuntary separation, compared to no permanent separation. Bootstrapping was applied to estimate confidence intervals (CI) for RR. All analyses were completed in R Studio 2022.07.1.

## Results

Of the 1832 participants in the OLAWIS1 and OLAWIS2 cohorts, 39 permanent separations that could not be accurately classified to either the health-related dimension or the voluntary/involuntary dimension were excluded from the analytic sample of 1793 observations.

The derived measure of accommodation adequacy classified 65.3% of participants as experiencing adequate accommodation (table 1). The majority of participants experiencing adequate accommodation received a modified work offer and were supported by a workplace representative in coordinating the implementation of the modified work offer. Additionally, 21.2% of participants classified as experiencing adequate accommodation, while not reporting receipt of a modified work offer, reported no difficulties in their return-to-work. Of the 34.7% of participants classified as experiencing inadequate accommodation, 55.4% reported receiving a modified work offer but did not report coordination assistance from a workplace representative and 44.6% did not receive a modified work offer and reported difficulties in their return-to-work.

**Table 1:** Classification of the adequacy of accommodation.^a^

		N		%
**Adequate accommodation**				
	Modified work offer with return-to-work coordination	920		
	No modified work offer, no return-to-work difficulties	248		
	Sub-total	1170		65.3
**Inadequate accommodation**				
	Modified work offer, no return-to-work coordination	346		
	No modified work offer, return-to-work difficulties	277		
	Sub-total	623		34.7
Total		1793		100.0

^a^ Classification missing for N=39 participants

Over the 18-month follow-up, the incidence of permanent separation was 30.7 per 100 workers (552 of 1793 participants). The incidence of permanent separation was equivalent in both the OLAWIS1 and OLAWIS2 cohorts, and between the shorter- and longer-duration sample groups. Among participants reporting permanent separation from the at-injury employer, 58.2% reported voluntary separations (188+133=321) and 48.1% reported involuntary separations (84+147=231) (table 2). Overall, 49.3% (272/552) of permanent separations were attributed by participants to impaired health and function arising from the disabling work-related injury/illness. Among voluntary separations, 58.6% (188/321) were attributed to impaired health and function from their work-related injury/illness. Among workers reporting involuntary separations, 36.4% (84/231) identified these health-related factors as a reason for the separation.

**Table 2:** Adequacy of accommodation by employment status at 18 months following disabling work-related injury or illness.^a^

		Employment status			Inadequate accommodation	
		N	% of column		N	% of row
**Permanent separation**		552	30.7		288	51.5
**Health-related separation**		272			165	60.7
	Involuntary	84			57	67.8
	Voluntary	188			108	55.9
**Not health-related separation**		280			123	43.9
	Involuntary	147			75	50.0
	Voluntary	133			48	35.3
**Continuing employment with the** **at-injury employer**		1241	69.3		341	27.4
Total		1793	100.0		623	34.7

^a^ Participants with other or unknown separation status (N=39) are excluded.

Prior to adjustment for worker and workplace characteristics, the incidence of permanent separation was substantially elevated among participants experiencing inadequate accommodation. Among the 69.3% of participants in continuing employment with the at-injury employer at the 18-month follow-up, 27.4% reported experiencing inadequate accommodation (table 2). In contrast, 51.5% of participants who permanently separated from the at-injury employer over the 18-month follow-up period experienced inadequate accommodation. Among workers who permanently separated, inadequate accommodation was more prevalent among workers reporting a health-related separation (60.7%) compared to workers whose separation was unrelated to health (43.9%).

A limited number of worker and workplace characteristics were weakly associated with experiencing inadequate accommodation. Exposure to inadequate workplace accommodation was more common among participants with lower levels of education, those born outside of Canada and among workers disabled by a sprain, strain, or dislocation injury. Among workplace characteristics, exposure to inadequate accommodation was more common among participants employed in small firms and among workers in non-unionized employment (table 3).

**Table 3:** Worker and workplace characteristics associated with accommodation adequacy. [SD=standard deviation.]

		Before inverse probability of treatment weighting					After inverse probabilityof treatment weighting			
		Adequate accommodation (N=1170)		Inadequate accommodation (N=623)			Adequate accommodation (N=1170)		Inadequate accommodation (N=623)	
		N (%)		N (%)	SD		N (%)		N (%)	SD
**Worker characteristics**										
Age group (years)										
	<30	149 (12.7)		81 (13.0)	0.039		12.7		12.6	0.002
	30–49	434 (37.1)		231 (37.1)			37.2		37.2	
	≥50	579 (49.5)		306 (49.1)			50.1		50.2	
	Missing	8 (0.7)		5 (0.8)			0.8		0.8	
Sex										
	Female	537 (45.9)		291 (46.7)	0.053		46.3		46.0	0.007
	Male	632 (54.0)		330 (53.0)			53.7		54.0	
	Missing	1 (0.1)		2 (0.3)			0.2		0.2	
Education										
	High school or less	305 (26.1)		195 (31.3)	0.090		28.6		28.8	0.004
	Some postsecondary	857 (73.2)		426 (68.4)			71.7		71.6	
	Missing	8 (0.7)		2 (0.3)			0.5		0.5	
Born in Canada										
	No	246 (21.0)		184 (29.5)	0.189		19.2		19.2	0.001
	Yes	915 (78.2)		436 (70.0)			77.8		77.8	
	Missing	9 (0.8)		3 (0.5)			0.6		0.6	
Urban/rural residence										
	Rural	220 (18.8)		120 (19.3)	0.013		19.1		19.0	0.001
	Urban	950 (81.2)		503 (80.7)			80.9		81.0	
Nature of injury										
	Sprain, strain, dislocation	514 (43.9)		288 (46.2)	0.138		44.5		44.5	0.011
	Superficial or open wound	135 (11.5)		74 (11.9)			11.6		11.7	
	Fracture	149 (12.7)		71 (11.4)			12.4		12.2	
	Internal injury	182 (15.6)		73 (11.7)			14.3		14.1	
	Other	39 (3.3)		24 (3.9)			3.5		3.6	
	Unknown	51 (4.4)		23 (3.7)			4.0		3.9	
	Missing	100 (8.5)		70 (11.2)			9.7		10.0	
Sample group										
	Short duration sample	507 (43.3)		226 (36.3)	0.143		40.8		41.1	0.010
	Long duration sample	663 (56.6)		397 (63.7)			59.2		58.9	
**Workplace characteristics**										
Union membership										
	No	565 (48.3)		343 (55.1)	0.123		51.9		52.2	0.006
	Yes	597 (51.0)		275 (44.1)			47.4		47.1	
	Missing	8 (0.7)		5 (0.8)			0.7		0.7	
Company size (employees)										
	<20	284 (24.3)		184 (29.5)	0.123		25.7		25.9	0.005
	20-99	394 (33.7)		192 (30.8)			32.2		31.9	
	≥100	446 (38.1)		219 (35.2)			38.1		38.1	
	Missing	46 (3.9)		28 (4.5)			4.0		4.1	
Employment type										
	Temporary	79 (6.8)		55 (8.8)	0.091		6.8		6.8	0.003
	Permanent	1090 (93.2)		566 (90.9)			93.2		93.2	
	Missing	1 (0.1)		2 (0.3)			0.1		0.1	
Economic sector										
	Goods producing	302 (25.8)		160 (25.7)	0.002		25.5		25.6	0.003
	Services	868 (74.2)		463 (74.3)			74.2		74.4	
	Missing	3 (0.3)		2 (0.3)			0.3		0.3	

Table 4 reports estimates of the RR of permanent separation associated with exposure to inadequate accommodation, accounting for worker and workplace characteristics associated with accommodation adequacy. The propensity score adjusted RR of a health-related separation associated with inadequate accommodation was substantial (2.72, 95% CI 2.20–3.73), greater than the RR of separations not related to health (1.68, 95% CI 1.38–2.21). The largest RR was attributed to involuntary separations that were health-related (4.73, 95% CI 2.98–9.03).

**Table 4:** Relative risk (RR) of permanent separation associated with exposure to inadequate accommodation.^a^ [CI=confidence interval.]

		Permanent Separations		Crude			Adjusted ^b^	
		N		RR	95% CI ^c^		RR	95% CI ^c^
**Health-related separation**		272		3.05	2.46–3.73		2.72	2.20–3.73
	Involuntary/health-related	84		5.35	3.29–9.03		4.73	2.98–9.03
	Voluntary/health-related	188		2.92	2.25–3.78		2.56	1.98–3.79
**Not health-related separation**		280		1.81	1.46–2.21		1.68	1.38–2.21
	Involuntary/not health-related	147		2.44	1.81–3.28		2.18	1.60–3.28
	Voluntary/not health-related	133		1.52	1.08–2.12		1.45	1.04–2.12

^a^ RR estimates for retired workers (N=32) and workers with other or unknown separation status (N=39) are excluded. ^b^ RR estimates adjusted for inverse probability of treatment weighting based on characteristics in table 3. ^c^ Confidence intervals estimated from bootstrapping.

## Discussion

In this cohort of disabled workers, the incidence of permanent separation over an 18-month period was approximately 30%. Among these separations, 58.2% were voluntary and 41.8% were involuntary. Overall, participants attributed 49.3% of permanent separations to impaired health and function arising from the disabling work-related injury/illness. During the course of recovery and return-to-work, 65.3% of participants experienced adequate workplace accommodation. The RR of a health-related separation arising from inadequate accommodation was substantial (2.72, 95% CI 2.20–3.73), exceeding the RR associated with separations not related to health (1.68, 95% CI 1.38–2.21).

The incidence of permanent separation in this cohort was approximately two times more frequent than would be expected among Ontario labor force participants (table 5). The majority of this elevated incidence in the OLAWIS cohorts are attributed to health-related separations. At ages 25–64 in Canada, approximately 15% of annual voluntary separations from employment are attributed to an illness or disability (1), generally mirroring the frequencies of HRES reported in cohort studies in high-income countries (9–11, 29). In the OLAWIS cohorts, 49.3% of permanent separations from employment with the at-injury employer were attributed to health conditions arising from a work-related injury or illness. The incidence of employment separations for non-health-related reasons in the OLAWIS cohorts (both voluntary and involuntary) was similar to the frequency among Ontario labor force participants.

**Table 5:** Incidence of job separations. [OLAWIS=Ontario Life After Work Injury Study; HRS=health-related separation]

		Ontario labour force, 2019						OLAWIS ^a^ cohort				
		Separations (000)		Employment (000)		Incidence per 100 employed		HRS		Non HRS		Total
		N		N		%		%		%		%
**Job leavers (voluntary separation)**												
Age (years)												
	15–24	221.1		1029.3		21.5						
	25–54	148.4		4812.5		3.1						
	55–64	75.4		1293.7		5.8						
Sub–total		444.9		7135.5		6.2		6.8		4.8		11.6
**Job losers (involuntary separation**												
Age (years												
	15–24	89.2		1029.3		8.7						
	25–54	144.4		4812.5		3.0						
	55–64	49.9		1293.7		3.9						
Sub-total		283.5		7135.5		4.0		3.0		5.4		8.4
Total separations						10.2		9.8		10.2		20.0

^a^ OLAWIS estimate adjusted to represent 12-month incidence (based on 18-month follow-up).

We note with some interest that, among workplace characteristics, neither economic sector nor employer size was strongly associated with the probability that a worker would receive adequate accommodation. Workers in unionized workplaces were more likely to receive adequate accommodation, reflecting a longstanding emphasis in organized labor to incorporate disability management standards in collective agreements.

Involuntary health-related separations, while not frequent, are an indication of the incidence of employer actions that violate legislated employment standards in this jurisdiction. Subject to some exemptions, Ontario employers have a 24-month obligation to re-employ workers disabled by a work-related injury or illness (30). In the OLAWIS1 and OLAWIS2 cohorts, approximately 4.6% of participants reported experiencing employment termination by the employer for reasons related to impairments of the workers’ health and function.

Population-based studies of HRES, based on administrative data sources or longitudinal surveys, have made important contributions to describing socioeconomic inequalities in HRES and have strong external validity (11, 29). The population-based surveillance of HRES based on sources of administrative information on employment status or income security benefits will typically not have information on workplace accommodation practices that can inform understanding of the influence of employer disability management practices in mediating the risk of health-related job loss (8, 10, 11, 31). One strength of this study was the inclusion of a measure of the adequacy of employer accommodation practices, which was found to be a strong predictor of the risk of health-related job loss. Optimal sources of information on workplace accommodation practices can be obtained from employer surveys (23, 25) or, as demonstrated in this study, from workers’ self-report.

Readers may have concerns about the reliability and validity of the measure of accommodation adequacy implemented in this paper. The measure is anchored to two distinct domains of disability management practice, work modifications and service coordination, for which a recent systematic review found strong evidence that the duration of work disability was significantly reduced in a systematic review of 36 medium- and high-quality studies (21). In an important fraction of disabling work-related injuries, recovery of function progresses quickly and there are limited needs for accommodation. While our study did not obtain information from workers on their perceived need for accommodation, we have proxied the status of low need for accommodation by implementing a composite measure based on respondents’ reports of no difficulties in the return-to-work process and the absence of an offer of modified work. The measure of accommodation adequacy is based on respondent self-reported recall over an 18-month period and may not be concordant with the perspective of the employer. As such, we acknowledge the potential for misclassification in the measure of accommodation adequacy. However, the robust association between accommodation adequacy and probability of permanent separation suggests any likely misclassification is not substantial.

We acknowledge a number of potential limitations in the study’s methods that should be considered when interpreting the study findings. It is possible that misclassification of the exposure measure – accommodation adequacy – was not equivalent among participants who separated from employment and participants who continued employment. In this condition of differential misclassification of exposure, there is a risk that the estimates of association between exposure and outcome are inflated relative to a true value. While we did not observe differences between participants and the claimant sample frame on age, sex, geographic location, industry, employer size and the duration of disability episode based on their administrative record, we cannot exclude the possibility that persons who had experienced inadequate accommodation and had permanently separated from employment were more likely to participate in the 18 month interview. If this selection bias was present, the estimated associations reported in this sample will be inflated relative to the population of injured workers.

Social insurance programs in developed countries typically include the provision of medical care benefits and wage replacement benefits for workers experiencing a work-related injury or illness. In addition to providing health care services to support the recovery of function, most work disability insurance schemes provide services to workers and employers to support work participation and return-to-work. This present study found that two thirds of participants received adequate accommodation in supporting their return-to-work following a disabling work-related injury or illness, which represents a substantial degree of compliance with established regulatory standards in this setting. However, given the risk of permanent separation among workers who did not receive adequate workplace accommodation, and evidence that there has been limited change over the past two decades in the proportion of employers who have implemented accommodation policies and practices (23), there is a clear need to devote efforts to expand the adoption of workplace accommodation practices in this setting.

A policy focus on supporting disabled workers to return-to-work with the at-injury employer provides important benefits to workers, employers, and disability insurance benefit schemes (25). The contribution of this study highlights the potential contribution of employers’ accommodation practices in enabling employment continuity over the long term. As demonstrated in this study and replicated in the majority of workplace-based cohort studies, workplace accommodation practices are a strong determinant of employment outcomes among workers with health impairments. Improved information on worker accommodation practices for representative samples of employers represents an important priority for future research.

## Supplementary material

Supplementary material
