# Supplementary material for: The adequacy of workplace accommodation and the incidence of permanent employment separations after a disabling work injury or illness
Source: Scand J Work Environ Health. 2024 Mar 27;50(3):208–17. doi: 10.5271/sjweh.4149 (PMC11117718; doi:10.5271/sjweh.4149)
Supplement: Supplementary material [file SJWEH-50-208-S001.pdf]

The adequacy of workplace accommodation and the incidence of permanent employment separations after a disabling work injury or illness[1]

by Cameron A Mustard, ScD,[2] Christa Orchard, MPH, Kathleen G Dobson, PhD, Nancy Carnide, PhD, Peter M Smith, PhD

1. *Supplementary material*
2. *Correspondence to: Cameron A Mustard, Institute for Work & Health, 400 University Ave, Suite 1800, Toronto, Ontario, Canada, M5G 1S5. [E-mail: cmustard@iwh.on.ca]*

**Why you are working with a different employer than 18 months ago when you were injured?**	Classification assigned at time of interview	Classification assigned following review of open text responses	Total
**Health-related Separation**			
**Involuntary separation / health-related**<br>The employer did not want worker to return to work, termination/dismissal by employer, employer would not modify duties, mistreatment and harassment	76	8	84
**Voluntary separation / health related**<br>Worker worried about re-injury, could not perform duties required in the work that was available	167	21	188
**Not Health-related Separation**			
**Involuntary separation / not health- related**<br>Employment contract had ended, or no work available, or laid off	122	25	147
**Voluntary separation / not health-related**<br>Quit to find a different job, left position for better opportunity, moved residence	89	44	133
**Total**	**454**	**98**	**552**
Other/Unknown	20	19	39
Retirement	32	0	32
Misclassified		78	
